# Drought Legacy Effects on the Composition of Soil Fungal and Prokaryote Communities

**DOI:** 10.3389/fmicb.2018.00294

**Published:** 2018-03-07

**Authors:** Annelein Meisner, Samuel Jacquiod, Basten L. Snoek, Freddy C. ten Hooven, Wim H. van der Putten

**Affiliations:** ^1^Microbial Ecology, Department of Biology, Lund University, Lund, Sweden; ^2^Sections of Microbiology and Terrestrial Ecology, Department of Biology, University of Copenhagen, Copenhagen, Denmark; ^3^Department of Microbial Ecology, Netherlands Institute of Ecology, Wageningen, Netherlands; ^4^Agroécologie, UMR1347, INRA Centre Dijon, Dijon, France; ^5^Department of Terrestrial Ecology, Netherlands Institute of Ecology, Wageningen, Netherlands; ^6^Theoretical Biology and Bioinformatics, Utrecht University, Utrecht, Netherlands; ^7^Laboratory of Nematology, Wageningen University, Wageningen, Netherlands

**Keywords:** climate change, soil, Birch effect, re-wetting, bacteria, fungi, microbial communities

## Abstract

It is increasingly acknowledged that climate change is influencing terrestrial ecosystems by increased drought and rainfall intensities. Soil microbes are key drivers of many processes in terrestrial systems and rely on water in soil pores to fulfill their life cycles and functions. However, little is known on how drought and rainfall fluctuations, which affect the composition and structure of microbial communities, persist once original moisture conditions have been restored. Here, we study how simulated short-term drying and re-wetting events shape the community composition of soil fungi and prokaryotes. In a mesocosm experiment, soil was exposed to an extreme drought, then re-wetted to optimal moisture (50% WHC, water holding capacity) or to saturation level (100% WHC). Composition, community structure and diversity of microbes were measured by sequencing ITS and 16S rRNA gene amplicons 3 weeks after original moisture content had been restored. Drying and extreme re-wetting decreased richness of microbial communities, but not evenness. Abundance changes were observed in only 8% of prokaryote OTUs, and 25% of fungal OTUs, whereas all other OTUs did not differ between drying and re-wetting treatments. Two specific legacy response groups (LRGs) were observed for both prokaryotes and fungi. OTUs belonging to the first LRG decreased in relative abundance in soil with a history of drought, whereas OTUs that increased in soil with a history of drought formed a second LRG. These microbial responses were spread among different phyla. Drought appeared to be more important for the microbial community composition than the following extreme re-wetting. 16S profiles were correlated with both inorganic N concentration and basal respiration and ITS profiles correlated with fungal biomass. We conclude that a drying and/or an extreme re-wetting history can persist in soil microbial communities via specific response groups composed of members with broad phylogenetic origins, with possible functional consequences on soil processes and plant species. As a large fraction of OTUs responding to drying and re-wetting belonged to the rare biosphere, our results suggest that low abundant microbial species are potentially important for ecosystem responses to extreme weather events.

## Introduction

Future climate is expected to include more variable drought and rainfall events (IPCC, 2012; Fischer and Knutti, 2016). These enhanced fluctuations will directly affect soil microbes that rely on water to fulfill their life cycles and activities (Vos et al., 2013). Fluctuations in soil moisture conditions influence the regulation of microbial activities such as respiration and growth (Manzoni et al., 2012; Meisner et al., 2015). For example, a pulse of respiration often occurs when water is added to dry soil (Birch, 1958; Kim et al., 2012). This is a general phenomenon that occurs in many regions in the world, such as arctic (Clein and Schimel, 1994; Meisner et al., 2017), temperate (Pulleman and Tietema, 1999; Meisner et al., 2013a), and arid regions (Miller et al., 2005; Chowdhury et al., 2011; Warren, 2014). The amount of water that is added during re-wetting determines the microbial response to the drying and re-wetting event (Orchard and Cook, 1983; Evans et al., 2014; Lado-Monserrat et al., 2014). Drying and re-wetting is considered a carbon loss from the microbial community (Schimel et al., 2007), as the carbon seems to be respired and not used for biomass incorporation via microbial growth (Blazewicz et al., 2014; Meisner et al., 2015). Whereas, the microbial growth, respiration rates and biomass are recovered 1 week after re-wetting (Lundquist et al., 1999; Meisner et al., 2013a), relatively little is known on how the composition of the microbial community recovers within weeks after re-wetting.

Drying and re-wetting can affect the composition of soil communities (Fierer et al., 2003; Barnard et al., 2013; Evans et al., 2014; Hartmann et al., 2017). Since terrestrial microbial communities are important regulators of many ecosystem services such as plant performance, bioremediation, and carbon cycling (Vidali, 2001; Schimel and Schaeffer, 2012; Philippot et al., 2013), soil moisture history may have important implications for these “higher-order” processes (Hawkes et al., 2017). For example, a past drought in the field can affect the bacterial composition when soil is exposed to drying and re-wetting events in the laboratory (Evans and Wallenstein, 2012). In addition, N mineralization and inorganic N availability is increased upon drying and re-wetting events (Meisner et al., 2013b). These so-called “legacy effects” provide a source of variation affecting microbes, but the extent and amplitude of their contribution to soil microbiome composition remains unclear.

Soil microbes have different strategies to cope with fluctuating moisture availability (Lennon et al., 2012). Bacteria have been shown to respond sensitively, tolerant or opportunistically to drying and re-wetting (Evans and Wallenstein, 2014). Bacterial sensitivity can be due to increased injuries in viable cells (Mackey and Derrick, 1984; Nocker et al., 2012), which may lead to a decrease in microbial activity (Kieft et al., 1987). Cell damage is difficult to repair during drought due to a decrease in microbial activity (Potts, 1994). As such, the fraction of “dormant-over-active” cells is likely to increase in drying soils (Manzoni et al., 2014) and more free niches will become available upon re-wetting. Microbes can tolerate drought when they produce protective molecules (Schimel et al., 2007), such as osmolytes (Warren, 2014). Opportunistic microbes can colonize free niches that become available upon re-wetting. Fast responding microbes (Placella et al., 2012) may affect the composition of the slower responders in the microbial community via priority effects (Fukami, 2015). Abundant microbes can take advantage of niches that become available after drought, however, sub-dominant and rare microbes may respond opportunistically as well (Aanderud et al., 2015; Lynch and Neufeld, 2015), so that growth rates of rare bacteria may not necessarily be different from those that are abundant (Kurm et al., 2017). The consequence of all these different response strategies is that extreme drought may imprint a legacy signature in the soil microbiome composition that can last for weeks after the end of the drought event.

Unlike bacteria, soil fungi are often less affected by drought due to their extended and exploratory hyphal structures (Bapiri et al., 2010; Yuste et al., 2011; de Vries et al., 2012a; Barnard et al., 2013). However, contrasting findings for the capacity of soil fungi to resist alterations in moisture conditions have been reported. The composition of fungal communities can differ between dry and wet conditions (Hawkes et al., 2011; Cregger et al., 2012; Acosta-Martínez et al., 2014; Barnes et al., 2018) and their biomass may increase, decrease, or remain unaffected by drought or irrigation (Scheu and Parkinson, 1994; Gordon et al., 2008; Haugwitz et al., 2014; Hartmann et al., 2017). Despite these contrasting findings, some fungal species are sensitive bioindicators of changes in soil moisture (Kaisermann et al., 2015). Therefore, fungal species may not only tolerate drought stress, but may respond sensitively or opportunistically (Crowther et al., 2014) just as bacteria.

Our aim was to test how extreme fluctuations in soil moisture content may result in a legacy in the composition of soil microbial communities. We identified legacy effects by applying high throughput molecular approaches. We tested the overall hypothesis that extreme drought and re-wetting events enact a legacy in soil via a changed composition of the microbial community after soil moisture conditions have been restored. More specifically, we predicted that: (1) an extreme drying and re-wetting event will decrease the number of abundant or sub-abundant OTUs, (2) fungal communities will also respond to changed moisture conditions by tolerant, opportunistic and sensitive response patterns, and (3) some rare microbes will increase in relative abundance. We further tested if the microbiome profiles were correlated to the previously measured soil processes, such as inorganic N concentration, N mineralization rates and respiration rates (see Meisner et al., 2013b for details on soil processes and nutrients). In order to test our hypotheses, we performed a mesocosm experiment and exposed soil to a 4 weeks drying treatment after which soils were either re-wetted to optimal (50% WHC, water holding capacity), or to saturated moisture conditions (100% WHC). The optimal soil moisture content is in between 50 and 70% WHC (Ilstedt et al., 2000; Setia et al., 2011). Soil was left to recover for 18 days, after which we extracted DNA and analyzed the composition of 16S rRNA gene and ITS amplicons.

## Materials and Methods

### Soil Origin

At November 3, 2009, soil was collected from five locations in the nature reserve Millingerwaard, which is located in the Gelderse Poort region along the Rhine River delta in the Netherlands (N51° 52.224′ E5° 59.494′). In the week before sampling, there was in total 22 mm of rain spread among the different days and the average air temperature was 11.5°C^1^. Coarse fragments and plant material were removed by sieving through a 10-mm sieve, and the remaining soil was homogenized and placed in the greenhouse where later on the experiment was carried out (see Meisner et al., 2013b for details). The soil is considered a Sandy soil (Meisner et al., 2011).

### Experimental Design

We tested how a history of an extreme drying and/or a re-wetting affected the composition of the microbial community 18 days after the soil was re-wetted to original conditions or re-wetted to saturation. Thereto, 32 microcosms were filled with 7L of soil (equivalent to 6000 g of dry soil) and left to settle for 2 weeks before the experiments started in a greenhouse with a temperature of 21°C (±2°C) for 16 h (±2°C) and 16°C (±2°C) for 8 h. The mesocosms were exposed to one of the four treatments: constant moisture content (Moist or “C”), drought stress for 28 days with re-wetting to 50% WHC (drought or “D”), extreme re-wetting to 100% WHC (extreme re-wetting or “ER”), or a combination of the drying and extreme re-wetting (drought and extreme re-wetting or “DR”). Soil samples were collected 18 days after the drought stress had stopped, at day 46 of the experiment. Soil was sampled with a small auger by taking ca. 100 gram (based on dry soil) from 5 to 7 places in the 7L mesocosms. Then, this soil sample was homogenized, after which a subsample was taken, put on ice and stored in the -80°C freezer as soon as possible after sampling. The non-frozen soil samples were used for the following measurements: Inorganic Nitrogen, Arginine Ammonification, basal respiration, substrate induced respiration, pH, ergosterol as fungal biomass indicator and total microbial biomass. The data have been published in a previous study (see Meisner et al., 2013b for details).

### DNA Extraction and Amplification

To characterize the bacterial and fungal soil communities we used barcode sequencing. Soil samples previously stored at -80°C were defrosted and from 250 mg of dried soil, total DNA was extracted for each sample using the PowerSoil DNA isolation kit (MO BIO Laboratories, Inc., Carlsbad, CA, United States) according to the manufacturer’s instructions. The composition of the prokaryotic community was determined by targeting a fragment of the 16S rRNA gene with amplicon sequencing. The PCR mixture contained 10 μl of 5 Prime Hot mastermix (QuantaBio), 1.25 μl BSA, 11.75 μl molecular grade water, 0.5 μl of 10 μm of the 515F, 0.5 μl of 10 μm 806R primers for amplification (Bates et al., 2011) and 1 μl template. The PCR conditions were the following: 94°C for 5 min, followed by 35 cycles of 94°C for 45 s, 50°C for 60 s, and 72°C for 1.30 min, followed by a final elongation of 10 min at 72°C. The composition of the fungal community was determined by targeting the ITS region. The PCR master mix contained 17.1 μl molecular grade water, 1 μl 5 μM DNtPs, 1 μl 25 mM MgCl_2_, 2.5 μl 10× PCR reaction buffer with MgCl_2_ (Roche), 1.25 BSA, 0.5 μl of 10 μm ITS4 (Ihrmark et al., 2012) primer and 0.5 μl of 10 μm fITS9 primer (Ihrmark et al., 2012), 0.15 μl of Taq DNA Polymerase (Roche) and 1 μl of template. PCR conditions were composed of an initial denaturation at 95°C for 10 min, followed by 35 cycles of denaturation at 94°C for 45 s, annealing at 54°C for 60 s and elongation at 72°C for 1.3 min followed with a final elongation at 72°C for 10 min. Both 16S and ITS PCR products were purified using the Agencourt AMPure XP PCR Purification kit (Beckman Coulter, Inc., Danvers, MA, United States). A PCR product/AMPure bead ratio of 1:0.7 was used. Elution is done with 25 μl preheated (55°C) Milli-Q water with an incubation time of 5 min. All other steps were performed according to the manufacturer’s protocol. After purification both amplicons were sequenced using the Illumina MiSeq platform for 300 bp paired-end reads.

### Annotation Pipeline

The raw paired-end Miseq data was processed into an annotated OTU table using the following pipeline. The RDP extension to PANDASeq (Masella et al., 2012) Assembler (Cole et al., 2014) was used to merge the raw reads using a minimum overlap of 150 bp and a minimum PHRED score of 25. Primer sequences were removed from the FASTQ files using Flexbar version 2.5 (Dodt et al., 2012). Sequences were converted to FASTA format and concatenated into one file. Sequence clustering was done using VSEARCH version 1.0.10 (Rognes et al., 2016) at 97% identity and using usearch_global with the default settings to map quality controlled reads against the OTU centroids. The strategy used for this was de-replication, sorting by abundance (at least 2 sequences) and clustering using the UCLUST smallmem algorithm (Edgar, 2010). Thereafter, chimeric sequences were detected and removed using the UCHIME algorithm (Edgar et al., 2011) implemented in VSEARCH. Lastly, taxonomic classification for each OTU was obtained by using the RDP Classifier version 2.10 using the bootstrap value of 80% and classification was done on full-length entries (Cole et al., 2014). The pipeline was made with Snakemake (Koster and Rahmann, 2012) as available at DOI: https://doi.org/10.5281/zenodo.597131 (de Hollander, 2016). This pipeline was also used for ITS with the following adjustments: (1) ITS2 regions where extracted using ITSx 1.0.11 (Bengtsson-Palme et al., 2013). (2) Using the UNITE database (Kõljalg et al., 2013) provided by RDP the sequences were classified. OTUs without affiliation to at least kingdom level were excluded from the downstream beta-diversity analysis as the carry little information.

### Sequences

Summary tables describing each sample are presented in Supplementary Tables S1, S2. The raw sequencing counts were used directly to estimate the sequencing depth completeness via rarefaction curves using the package *vegan* in R (Supplementary Figure S1). Although amplicon profiles didn’t achieved asymptotic approximation (Supplementary Figure S1), sequencing depth was sufficient to analyze the core majority of this soil microbial diversity with a satisfactory rarefaction threshold (Schöler et al., 2017). There were on average 23808 OTUs per sample for 16S (Supplementary Table S1) of which three were excluded from further analysis due to too few counts or too low richness. For ITS, there were on average 4106 OTUs per sample (Supplementary Table S2) of which one sample was excluded from further analysis due to too few counts. The R software (R Core Team, 2017) and PAST software were used (Hammer et al., 2001). Raw fastq files are available on the European Nucleotide Archive (ENA) and have accession number PRJEB23318.

### Biostatistics

#### Richness and Evenness Analysis

Diversity indices were analyzed as described in previous work (Jacquiod et al., 2016; Nunes et al., 2016). The Shannon diversity index (*H*) was calculated as H = -∑ p_i_ ln (*p*_i_), where *p*_i_ is the proportional abundance of OTU *i* in the mesocosms. Shannon diversity indices (H, Gaussian glm model fit) and sample richness (R, Poisson glm model fit) were calculated on rarefied data at 50,000 counts per sample for 16S rRNA gene profiles and 12,000 counts per sample for ITS profiles in order to avoid biases that may come from uneven numbers. Univariate statistical analysis was done in R version 3.1.1. We used the *multcomp* package version 1.3.6 (Hothorn et al., 2008) for the ANOVA corrected with Tukey *post hoc* test.

#### Multivariate Analysis and Constrained Ordination

The multivariate analysis was done with the raw and non-rarefied contingency tables using the R software version 3.0.2 with the functions vegdist, hclust, rda, Adonis in package *vegan* and function dudi.pca, bca, randtest, s.class, in package *ade4*. A log10 transformation was needed to improve normality of data. Principal Component Analysis (PCA) was performed after center-scaling normalization. A pattern search was applied to the original PCAs by grouping replicates together in order to perform a Between Group Analysis (BGA). The statistical significance of the selected grouping factor was tested with a Monte-Carlo simulation involving 10,000 permutations. Complementing PERMANOVA tests were performed on the Euclidean distance profiles using 10,000 permutations in order to assess differences across the four legacies. An ANOVA was performed with phyla as response variables and treatment as explanatory variable. Differences between the four treatments were identified using a *post hoc* Tukey–Kramer test and false discovery rate multiple correction test (functions glm, cld, gltht in package *multcomp*, FDR, *P* < 0.05). Redundancy analysis (RDA) were performed as described before (Nunes et al., 2016) using function rda in vegan package, after center-scaling normalization for both ITS and 16S rRNA gene amplicon profiles using the following explanatory variables: four moisture legacies as well as the soil measurements, inorganic nitrogen, arginine ammonification, basal respiration, substrate induced respiration, pH, ergosterol, total microbial biomass (see for details: Meisner et al., 2013b). A permutation test was performed using 999 permutations to test the robustness of the model for discrimination of 16S or ITS profiles.

#### Definition and Validation of Legacy Response Groups (LRGs)

We classified microbial OTUs into Legacy Response groups (LRGs), which are defined as groups of organisms with similar response to a change in their environment. Here, we apply this concept to define groups of OTUs that may respond differently to a legacy effect of drought. LRGs were extracted from microbiomes as described previously (Nunes et al., 2016; Jacquiod et al., 2018). Fine changes in the soil microbiomes at the OTU level were extracted using negative binomial distribution and generalized linear model (nbGLM) (Robinson et al., 2010; Schöler et al., 2017). Significance of OTU changes was inferred with a quasi-likelihood *F*-test (QLF) under *post hoc* false discovery rate multiple correction test with the package *edgeR* in R (FDR, *p* < 0.05) (Robinson et al., 2010). This method has been suggested recently as one of the most accurate ways to extract significantly responding OTUs by minimizing the risk of error (Thorsen et al., 2016). LRGs aggregating OTUs based on similar response patterns were identified using hierarchical clustering and heatmaps representation, followed by Monte-Carlo simulation for statistical validation. Enrichment of phylogenetic groups in LRGs was tested using a hypergeometric test. We asked for each phylogenetic level and each phylogenetic group if we found more of that specific group in the LRG than expected by chance depending on the size of the LRG and on the occurrence of that group in the total sample. We determined the proportion of OTUs in LRGs belonging to rare microbes. Thereto, we considered the OTUs that had a relative abundance below 0.01% as rare (Galand et al., 2009).

## Results

### Evenness and Richness

**FIGURE 1:**
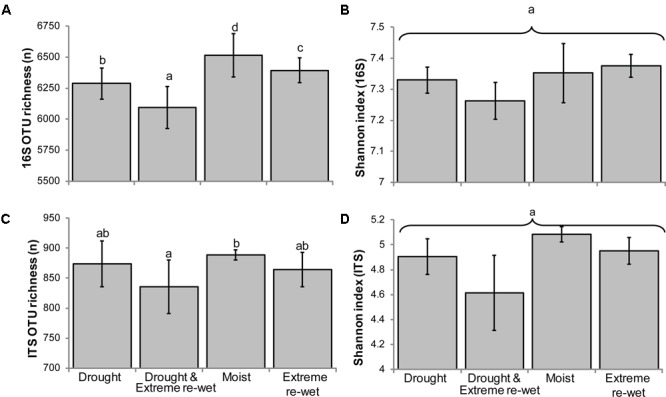
Richness and evenness of ITS and 16S rRNA gene amplicon profiles according to drying and re-wetting treatments (Average ± SEM). The chart displays the richness **(A)** and the Shannon indices **(B)** for 16S rRNA gene profiles and the richness **(C)** and Shannon indices **(D)** for ITS. Letters denote differences at *P* < 0.05 for a *post hoc* Tukey HSD correction test. ANOVA was run with the Poisson distribution for the richness.

The legacy of drying and/or extreme re-wetting appeared as decreased OTU richness for both ITS and 16S-based profiles, but not the evenness (**Figure 1**). In both profiles, the lowest richness was observed when soils were exposed to drying and extreme re-wetting, while the highest richness was obtained in the moist control soil. The 16S rRNA gene profiles showed a gradual decrease in richness, in descending order with the following soil history: moist control, extreme re-wetting, drying, drying and extreme re-wetting.

### Constrained Ordination and Redundancy Analysis

**FIGURE 2:**
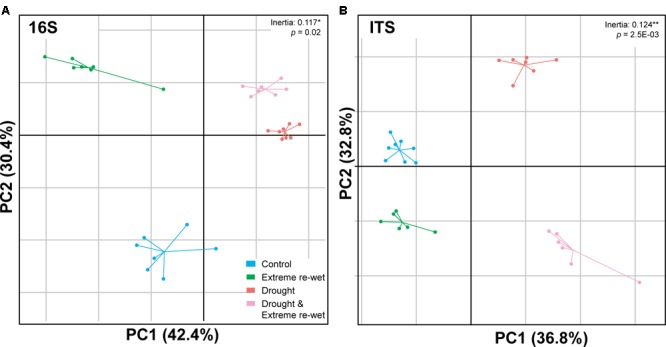
Between Group Analysis (BGA) of the soil microbial communities. The figure shows constrained principal component analysis (PCA) of the microbiome profiles after applying sample grouping, according to replicates for four different moisture treatments for 16S rRNA gene profiles **(A)** and ITS profiles **(B)**. Non-random distribution of the BGA grouping was tested using a Monte-Carlo simulation with 10.000 permutations (ITS: *p* = 2.5E–03^∗∗^; 16S: *p* = 0.02^∗^).

**FIGURE 3:**
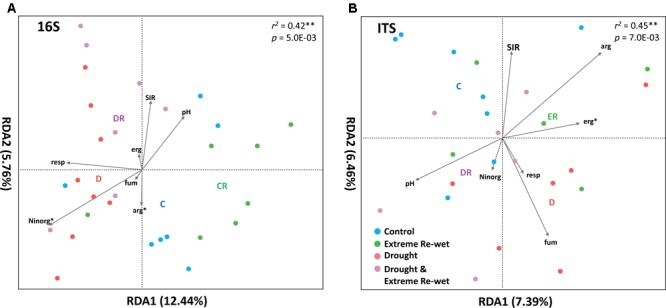
Redundancy analysis of prokaryotes **(A)** and fungi **(B)** with as explanatory variables: Moist, Extreme Re-wetting, Drought or Drought and Extreme re-wetting. In addition, we have used the following continues explanatory variables: Ninorg (N inorganic), resp (Basal Respiration), SIR (Substrate Induced respiration), erg (ergosterol content, which is a measure of fungal biomass), Fum (total biomass measured with fumigation extraction), arg (arginine ammonification). The *R*^2^ indicated the fit of the model and the *P*-value the significance of all axis (tested with a permutation test, *n* = 999).

The ITS and 16S profiles were separated in soil with a history of drying and/or extreme re-wetting (**Figure 2**). However, there was also variation within the different treatments as demonstrated by the spread in the replicates of the control and extreme re-wetting for 16S, and drying and extreme re-wetting for the ITS. Drought legacy explained 8.4% of the PERMANOVA variation in 16S profiles and 6.1% of the variation in ITS profiles, whereas the legacy of extreme re-wetting only explained 4.3% of the variation in ITS and none in 16S (**Table 1**). Redundancy analysis revealed that the microbiome profiles of 16S correlated with inorganic N content and soil respiration (**Figure 3A**). In addition, the microbiome profiles for ITS correlated with the amount of ergosterol (**Figure 3B**).

**Table 1:** PERMANOVA on Euclidean distance matrix of the microbiomes.

PERMANOVA (Euclidean distance, 10000 permutations)							
16S rRNA gene profiles				ITS profiles			
Factor	*r*^2^	*p*	Signif.	Factor	*r*^2^	*p*	Signif.
1: Legacy	0.084	10E-05	^∗∗∗^	1: Legacy	0.061	10E-05	^∗∗∗^
2: Re-wetting	0.040	0.13	–	2: Re-wetting	0.043	0.041	^∗^
1:2	0.035	0.44	–	1:2	0.039	0.27	–
Residual	0.83	–	–	Residual	0.85	–	–

*The respective effects of legacy, re-wetting and their interaction were assessed. Statistical significance was inferred based on 10,000 permutations (^∗∗∗^*p* < 0.001; ^∗^*p* < 0.05).*

### Legacy Response Groups – 16S rRNA Genes

**FIGURE 4:**
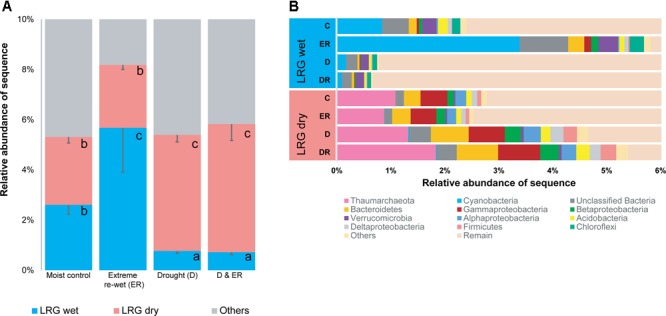
The relative abundance of the Legacy Response Groups (LRGs) for 16S amplicons **(A)** and the taxonomic affiliation of each group **(B)**. Letters denote differences among the four treatments within one LRG at *P* < 0.05 (ANOVA, Tukey HSD *post hoc* test).

Two LRGs were identified for 428 prokaryote OTUs, representing 5–8% of the total number of sequences, and 1.8% of the total number of OTUs in this study (428/23880). Prokaryote OTUs were either decreased in relative abundance in soil with a history of drought (LRG wet) or increased in relative abundance in soil with a history of drought (LRG dry; **Figure 4**; see heat maps in Supplementary Figure S2). OTUs belonging to the phylum Cyanobacteria, Chloroflexi, and Verrucomicrobia were enriched in LRG wet (Supplementary Table S3 and **Figure 4**) and OTUs belonging to the phylum Thaumarchaeota, and Proteobacteria were enriched in LRG dry (Supplementary Table S4 and **Figure 4**). OTUs belonging to Bacteroidetes were enriched in both LRG dry and LRG wet. This is mostly driven by the response of Sphingobacteriaceae and Flavobacteriaceae in LRG wet and by the response of Chitinophagaceae and Cytophagaceae in LRG dry. Responding OTUs were less rare than the total amount of rare OTUs in the community, which was 92% of the OTUs. For LRG wet, ca. 80% of the OTUs belonged to the rare biosphere in the non-drought soil and ca. 92% in the dried soil (Supplementary Figure S3A). For LRG dry, ca. 80% belonged to the rare biosphere in the non-drought soil and this decreased to ca. 70% in the dried soil (Supplementary Figure S3B). As such, some OTUs that were more abundant decreased in relative abundance when exposed to drought in LRG wet whereas rare OTUs become more abundant in LRG dry (Supplementary Figure S3). Although the initial drought had the most structuring effect on OTUs belonging to the two responding groups, the extreme re-wetting also affected some OTUs when further analyzing if there were LRG within soils with or without a history of drought (see Supplementary Figure S4).

### Legacy Response Groups – ITS

**FIGURE 5:**
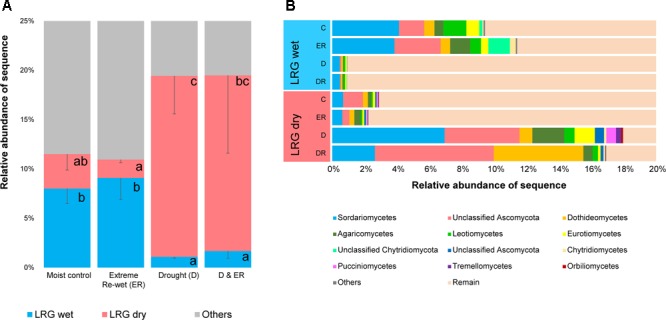
The relative abundance of the Legacy Response Groups for ITS amplicons **(A)** and the taxonomic affiliation of each group **(B)**. Letters in the left panel denote differences among the four treatments within one LRG at *P* < 0.05 (ANOVA, Tukey HSD *post hoc* test).

Two LRGs were also identified for 113 fungal OTUs, accounting for ∼20% of ITS sequences, and 12.3% of the total number of OTUs (**Figure 5**, see heat maps in Supplementary Figure S5). Fungal OTUs were either decreased in relative abundance in soils with a history of drought (LRG wet) or increased in relative abundance by drought legacy (LRG dry). OTU belonging to the Ascomycota were enriched in the LRG dry group (Supplementary Table S5) although there were also responding OTUs that belonged to the Ascomycota that decreased in relative abundance due to drought history. Sordariomycetes, Diaporthales, unclassified Sordariomycetes, Sordariales, Eurotiomycetes and Dothideomycetes were the most responsive Ascomycota within LRG dry as OTUs belonging to these orders were increased in relative abundance by drought legacy. Sordariomycetes and Leotiomycetes were orders that were decreased in relative abundance by drought legacy. There was no clear linkage between others OTUs and their phylogenetic origins. About half the OTUs can be considered rare in the moist control soils, and a higher number of these rare OTUs were observed in the soil exposed to drought for LRG wet (Supplementary Figure S6). However, these responding OTUs were less rare than the total amount of rare OTUs in the community, which was 77%. As previously seen for prokaryotes, the extreme re-wetting had an effect within the overruling legacy effect of drought (Supplementary Figure S7).

## Discussion

### Responses Are Spread Among Phyla

Our results supported our overall hypothesis that extreme simulated drying and/or extreme re-wetting leave a legacy in the composition of the soil microbial community after abiotic conditions have been restored. We found that soils with a history of drying and/or extreme re-wetting differed in composition of prokaryotic and fungal communities. Fungi and prokaryotes responded not only sensitively, but also resistant and opportunistically to a history of drought, which has also been shown for soils exposed to multiple drying and re-wetting cycles (Evans and Wallenstein, 2014). OTUs were grouped in distinct LRGs, regardless of their phylogenetic origin, as the responses of bacteria and fungi were spread among different phyla, classes, and orders. For example, Bacteroides OTUs decreased and increased in relative abundance in soil with a history of drought, which is similar as observed in other studies that measured the response directly to drought (Evans and Wallenstein, 2014). In addition, Chloroflexi can decrease in relative abundance under drought conditions (our study), increase in relative abundance under aridity (Maestre et al., 2015), or has a mixed response to altered moisture conditions (Hartmann et al., 2017). This would suggest that adaptation to extreme moisture fluctuation in soils is likely to have evolved independently in different microbial phyla, leading to the parallel selection and development of similar responders in separated phyla. One possible explanation for responses being spread among phyla is that microbes with oligotrophic and copiotrophic strategies have been suggested to be widespread among phylogenetic groups (Ho et al., 2017). This suggests that resources becoming available for the microbial communities upon re-wetting can be used by a wide range of species with a copiotrophic strategy that belong to different phyla. The initial fast responding species may be important for the composition of slower responding species (Fukami, 2015). As such, the initial copiotrophic strategy may be important for the successional outcome of the microbial re-colonization of empty niches that became available after the drought event.

### Prokaryotes Responses to Drought Legacy

Our results partly support our first hypothesis that the history of drying and/or extreme re-wetting event decreases the relative abundance of sub-dominant taxa, as this was only the case for bacteria and fungi belonging to the first response group (LRG wet). Cyanobacteria seem to have responded sensitive to drought, because they decreased in soil with this legacy, which is in line with studies on Cyanobacteria in soil crusts (Williams et al., 2008; Hagemann et al., 2017). As Cyanobacteria might have a slow recovery upon re-wetting (Williams et al., 2008), they will most likely not have recovered 3 weeks after the drought period had stopped.

Certain bacterial groups increased in abundance in soils with a history of drought. There are three reasons why some OTUs increased in relative abundance in soil with a history of drought (LRG dry). First, OTUs belonging to certain Prokaryotes may have resisted the drought stress. For example, the most abundant group of Archaea, Thaumarchaeota, increased in relative abundance in soil with a history of drought. This group has been found in extreme environments (Stieglmeier et al., 2014) and can be highly abundant in dessert soil (Shi et al., 2016). Second, bacteria with an opportunistic strategy have likely belonged to the fast responders that increased their abundance after re-wetting (Placella et al., 2012). For example, OTUs that belonged to Alpha-, Beta- and Gammaproteobacteria increased in relative abundance in soil with a history of drought (**Figure 3**). These classes have been identified as typical copiotrophs with high growth rates (Philippot et al., 2010). Third, some groups of bacteria may have responded via microbial facilitation mechanisms. For example Cyanobacteria and Acidobacteria can produce extracellular polysaccharides that can create moist micro-niches that may benefit other bacteria during drought conditions (Kielak et al., 2016). As there were some OTUs from both groups present in soil that have experienced a drought legacy (**Figure 3**), these moist micro-niches may have helped other bacteria to survive the drought period.

### Response of Fungi to Drought Legacy

Our results contrast previous reports showing that fungi are more resistant to drought than bacteria (Barnard et al., 2013; Meisner et al., 2013a). Instead, a short-term history of drought remained in the soil as a legacy effect in the composition of the fungal community. The fungi responded sensitive, tolerant or opportunistic to drying and re-wetting. Long-term drought treatments have been observed to affect the composition of fungi (Sayer et al., 2017). In addition, the composition of fungal community is shaped by rainfall amounts (Hawkes et al., 2011), and can differ between wet and dry seasons (Cregger et al., 2012; Acosta-Martínez et al., 2014). As such, the fungal communities differ from wet to drought conditions and remain present when soil has a short-term drought history. Responding fungal OTUs occurred in Ascomycota; in particular the Sordariomycetes and Dothideomycetes appeared to be major responders to drought history (**Figure 4**). Some fungi can likely respond to small moisture fluctuations, because they can use an opportunistic strategy thereby changing the composition of the fungal community (Kaisermann et al., 2015).

### Drought and Microbiome Response to Extreme Re-wetting

The history of extreme re-wetting could be identified for fungi and prokaryotes when soils with a history of drought were analyzed separately from the other soils (Supplementary Figures S4, S7). This suggests that drought has a major contribution to overall changes in the composition of the microbial community. As a consequence, a second dichotomy was identified between OTUs reacting to extreme re-wetting, leading to the identification of two additional sub-response groups. Gamma- and Betaproteobacteria were increasing or decreasing in relative abundance when re-wetting to maximum moisture. This is consistent with an earlier study where Proteobacteria have been identified as a group that clearly responded to re-wetting (Evans et al., 2014). The increase in relative abundance of Cyanobacteria and decrease in Firmicutes was clearly linked to the maximum water addition in soils without a history of drought. The same trends were observed for fungi, although the phylogenetic linkage was not as obvious as for bacteria since a wide diversity of lineages were always affected positively and negatively by the extreme re-wetting.

### Rare Microbes Belong to Responding OTUs

Our work supports the third hypothesis that some rare microbes will increase in relative abundance due to drought legacy. This supports the view that there are a high number of rare taxa in the microbial community, which is common in soil (Lynch and Neufeld, 2015). Members of the rare biosphere can be highly active and contribute to processes in soil even if they have low-ranked abundance (Pester et al., 2010; Pedrós-Alió, 2012). A large fraction of the rare bacteria can respond within days after re-wetting (Aanderud et al., 2015). In addition, the growth rates of low-abundant microbes are not necessarily different from abundant ones (Kurm et al., 2017). As such, the history of drought may potentially lead to altered composition of the microbial community through effects on rare members of the soil microbial community, without obvious consequences on the overall evenness indices.

### Ecosystem Consequences of Drought Legacies

Our results suggest that drying and re-wetting events can cause a legacy effect in the microbial communities due to a direct effect of moisture. The consequences of altered composition of microbial community due to a history of drought can modify the performance of plant species in communities when other abiotic conditions were kept constant (Meisner et al., 2013b). In addition, changes in soil microbial communities due to drying and/or extreme re-wetting influenced fitness of a rapidly reproducing plant species (Lau and Lennon, 2012). Moreover, changes in soil microbial communities due to drought legacies can affect plant-soil feedbacks (Kaisermann et al., 2017). As such, extreme weather effects on composition of the microbial community may also have the potential to influence the composition and functioning of plant species in terrestrial ecosystems.

Fluctuations between drying and re-wetting do not only affect the composition of soil microbial communities, but can also boost soil fertility by increasing nitrogen availability for plants (Birch, 1958; Jarvis et al., 2007; Meisner et al., 2013b). Previous work showed that drought legacy effects resulted into increased available nitrogen and soil respiration rates (Meisner et al., 2013b), which also seems to be correlated with composition of the prokaryotes (**Figure 3**). On the one hand, this indicates that the abiotic changes during drought affected nitrogen availability and soil functions and therewith the composition of the microbial community. On the other hand, one of the major changes during drought is a decrease in microbial activity (Meisner et al., 2017) and biomass (Kieft et al., 1987), which increases the number of niches available for microbes to recolonize upon re-wetting. Increased nitrogen availability has also been suggested to positively correlate with plant growth performance following a period of drought (Lebedjantzev, 1924; de Vries et al., 2012b). For example, pre-season drought effects may increase invasiveness of non-native plants that shift range as a consequence of climate warming (Meisner et al., 2013b). Drought can also affect the composition of plant species directly (Kardol et al., 2010). Fluctuations in drying and extreme re-wetting events and magnitudes have also been suggested to affect soil microbial communities via changes in plant composition (de Vries et al., 2012b; Evans et al., 2014). Future research is needed in order to reveal how drought legacies can affect the interactions between plants and soil microbes at different temporal, for example successional, stages of ecosystem development. Therefore, we conclude that effects of extreme drying and re-wetting may remain in the soil as a legacy effect in the composition of the microbial community. These legacy effects may explain why the composition of terrestrial plant communities change even once the extreme weather event ends (Meisner et al., 2013b).

## Data Accessibility

Sequences are deposited on the European Nucleotide Archive (ENA) and have accession number PRJEB23318.

## Author Contributions

AM and WvdP designed the experiments. FtH performed the lab analysis. BS and SJ analyzed the data. AM wrote the first draft of the manuscript and all other authors were involved in revising the manuscript text. All authors approved the submitted version of the manuscript.

## Conflict of Interest Statement

The authors declare that the research was conducted in the absence of any commercial or financial relationships that could be construed as a potential conflict of interest.

## Footnotes

1 www.knmi.nl
