# Supplementary material for: Drought Legacy Effects on the Composition of Soil Fungal and Prokaryote Communities
Source: Front Microbiol. 2018 Mar 7;9:294. doi: 10.3389/fmicb.2018.00294 (PMC5845876; doi:10.3389/fmicb.2018.00294)
Supplement: Supplementary file 1 [file Presentation_1.pdf]

Supplementary Material

Drought legacy effects on the composition of soil fungal and prokaryote communities

Annelein Meisner*. Samuel Jacquiod, Basten L. Snoek, Freddy ten Hooven, Wim H. van der Putten

*** Correspondence:** Corresponding Author: Annelein.Meisner@biol.lu.se

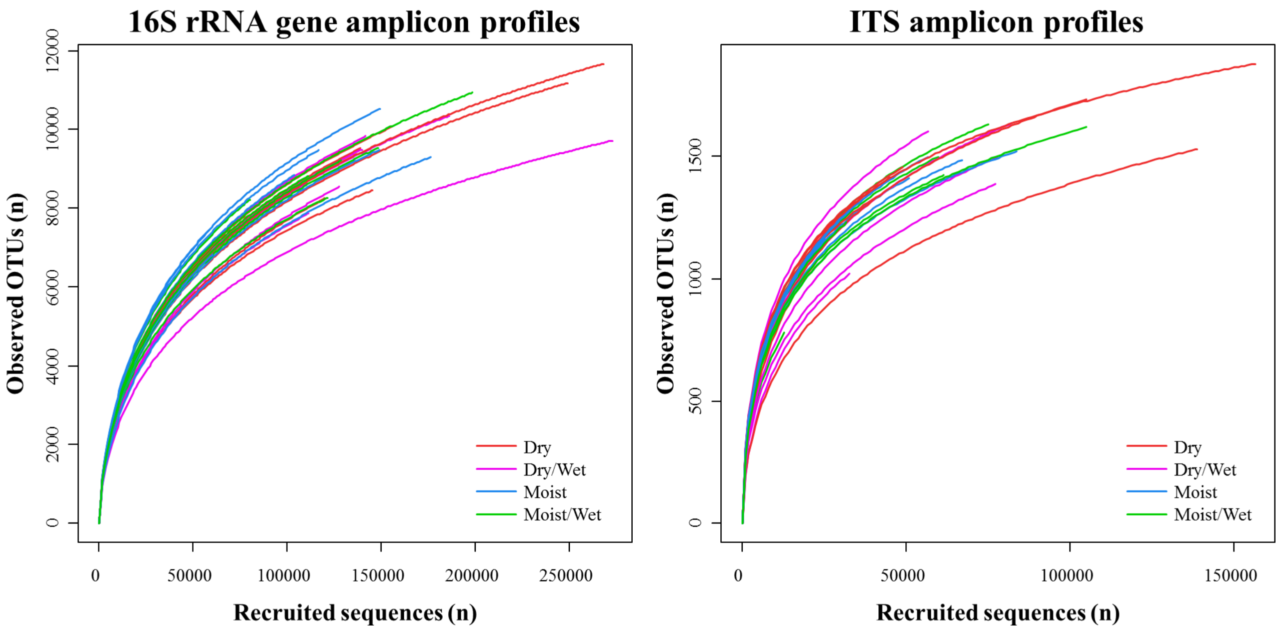

Figure S1: Rarefaction curves showing the OTU richness detection in each sample as a function of recruited sequences. Each panel is respectively showing the corresponding legacy of drying (dry) and extreme re-wetted (wet) samples for 16S rRNA gene profiles (left) and ITS (right).

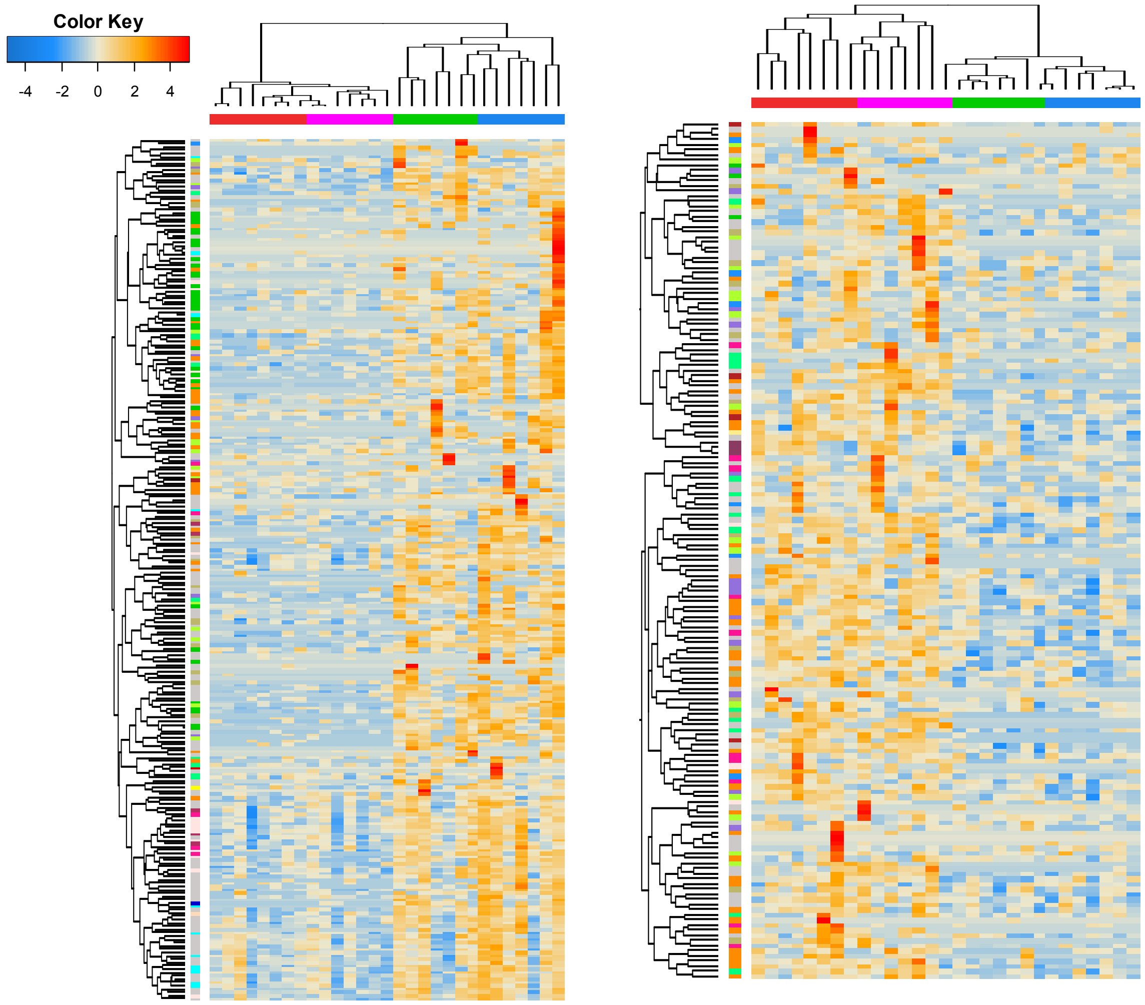

PhyClass	Rcolors
Acidobacteria	deeppink
Actinobacteria	dodgerblue
Alphaproteobacteria	mediumpurple
Bacteroidetes	darkorange
Betaproteobacteria	springgreen
candidate_division_WPS-1	peachpuff
candidate_division_WPS-2	peachpuff
Chlamydiae	yellow
Chloroflexi	cyan
Cyanobacteria	green3
Deltaproteobacteria	darkkhaki

PhyClass	Rcolors
Euryarchaeota	mediumblue
Firmicutes	firebrick
Gammaproteobacteria	greenyellow
Gemmatimonadetes	lightgoldenrod1
Parcubacteria	red3
Planctomycetes	maroon
Unclass_Bacteria	snow3
Unclass_Proteobacteria	snow3
Unclass_Root	snow3
Unclass_Archaea	snow3
Verrucomicrobia	mistyrose
Thaumarchaeota	hotpink4

Figure S2: Heat maps of Responding OTUS to drought legacy for 16S. Left are OTUs belonging to legacy response group 1 and the right panel shows OTUs belonging to legacy response group 2. The color code above the heat maps indicates the treatment with red drying; pink drying and extreme re-wetting; green: moist control; and blue extreme re-wetting.

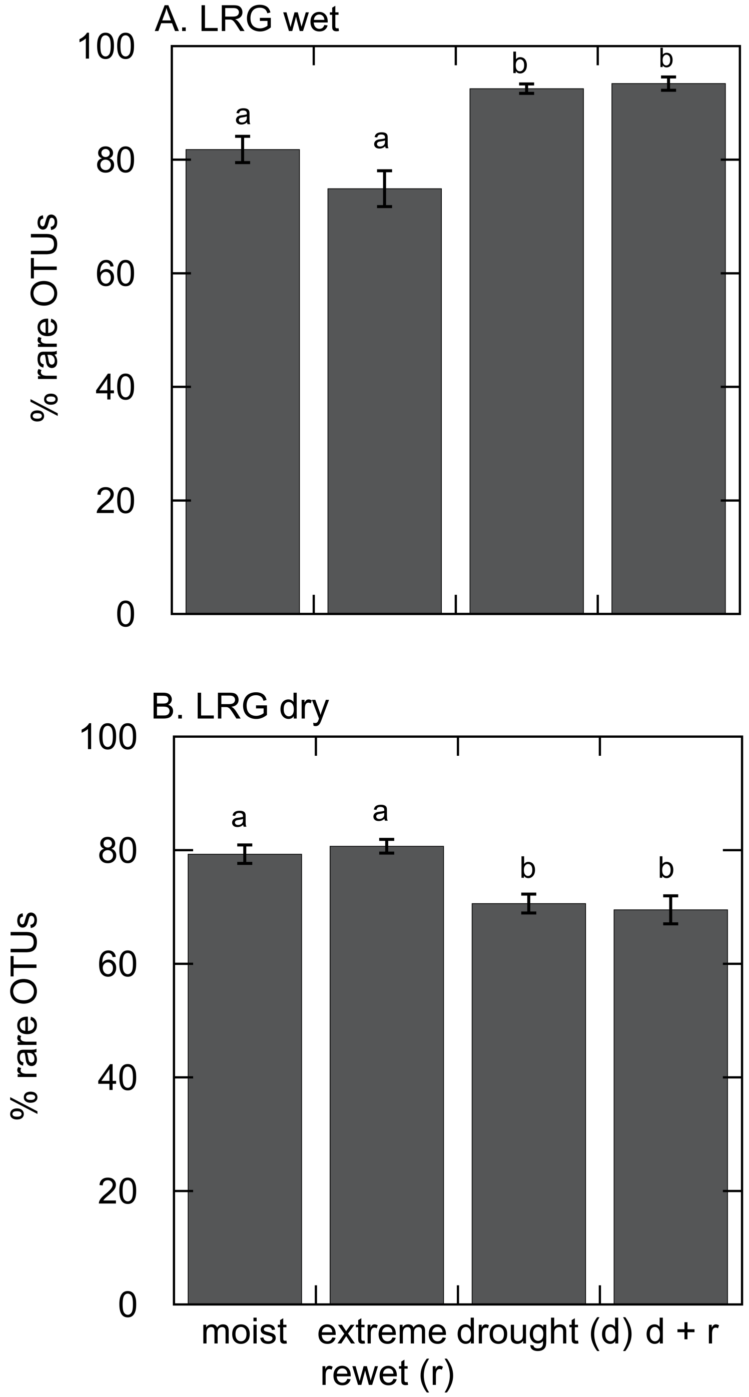

Figure S3: rare abundant OTUs in the LRG wet (a) and LRG dry(b) for the 16S amplicons. Letters denote significant differences between treatments after a Tukeys posthoc test ($P<0.05$).

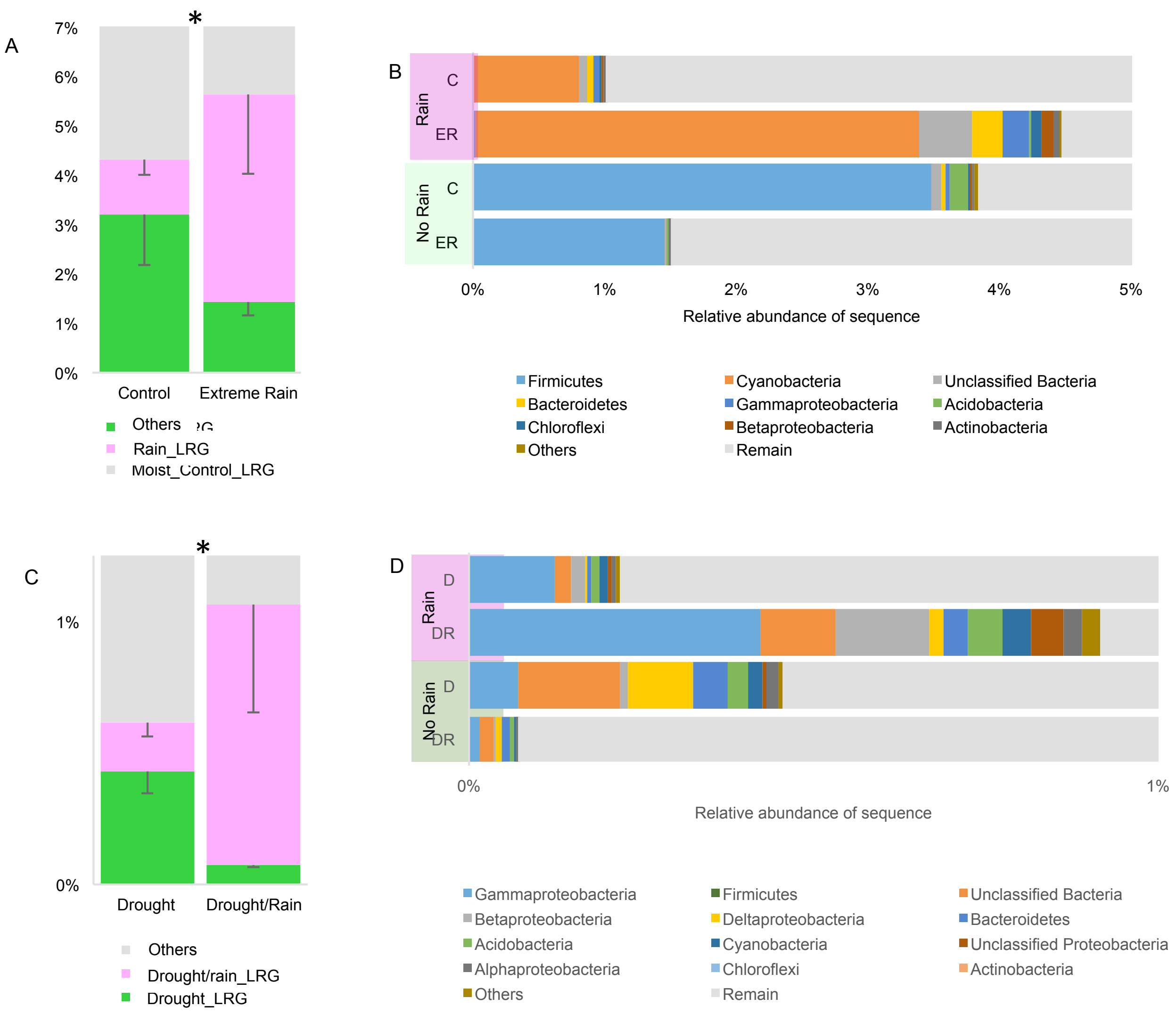

Figure S4: Legacy Response Groups were performed separately for Moist Control versus extreme rain (extreme re-wetting) (A+B) and Drought versus Drought+Rain (extreme re-wetting) (C+D). The relative abundance of OTUs that belong to the Response groups (A,C) and the taxonomical affiliation are presented (B,D). *indicates a difference between the groups at $P<0.05$.

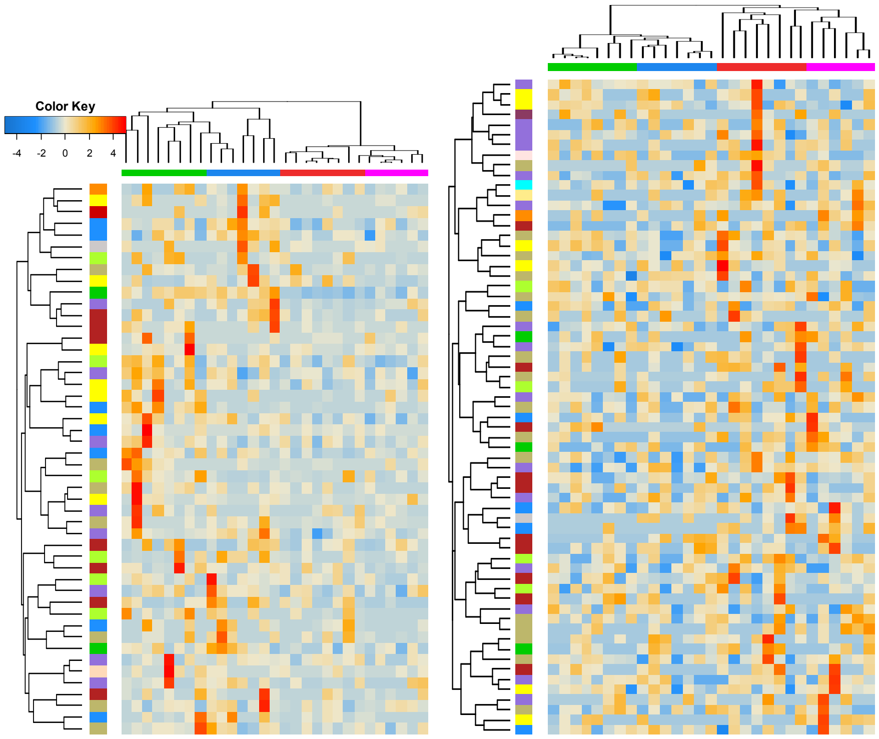

Class	color
Agaricomycetes	firebrick
Archaeorhizomycetes	red3
Ascomycota_Unident	darkkhaki
Basidiomycota_Unident	snow3
Chytridiomycetes	green3
Chytridiomycota_Unident	green3
Dothideomycetes	dodgerblue
Eurotiomycetes	greenyellow
Glomeromycota_Unident	lightgoldenrod1
Leotiomycetes	yellow
Microbotryomycetes	peachpuff
Orbiliomycetes	hotpink4
Pezizomycetes	darkorange
Pucciniomycetes	mistyrose
Sordariomycetes	mediumpurple
Tremellomycetes	cyan
Unclass_Ascomycota	darkkhaki
Unclass_Basidiomycota	snow3

Figure S5: Heat maps of Responding OTUS to drought legacy for ITS. Left are OTUs belonging to functional response group 1 and the right panel shows OTUs belonging to functional response group 2. The color code above the heat maps indicates the treatment with red drought; pink drought + extreme re-wetting; green: moist control; and blue extreme re-wetting.

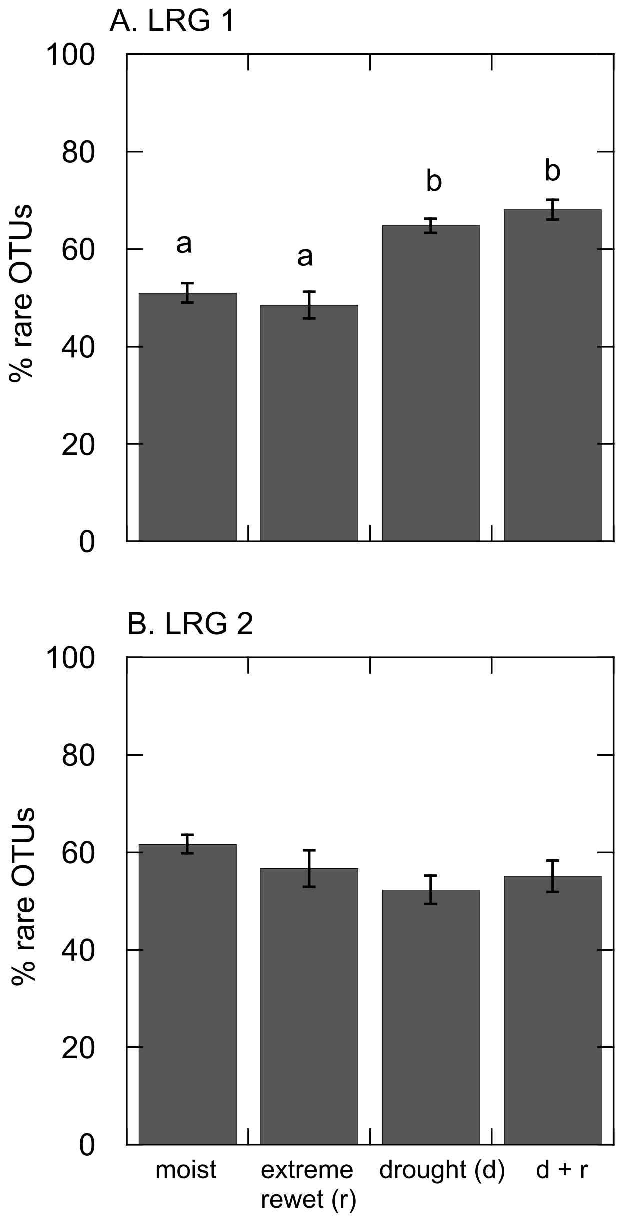

Figure S6 rare abundant OTUs in the LRG 1 (a) and LRG 2(b) for the ITS amplicons. Letters denote significant differences between treatments after a Tukeys posthoc test (P<0.05).

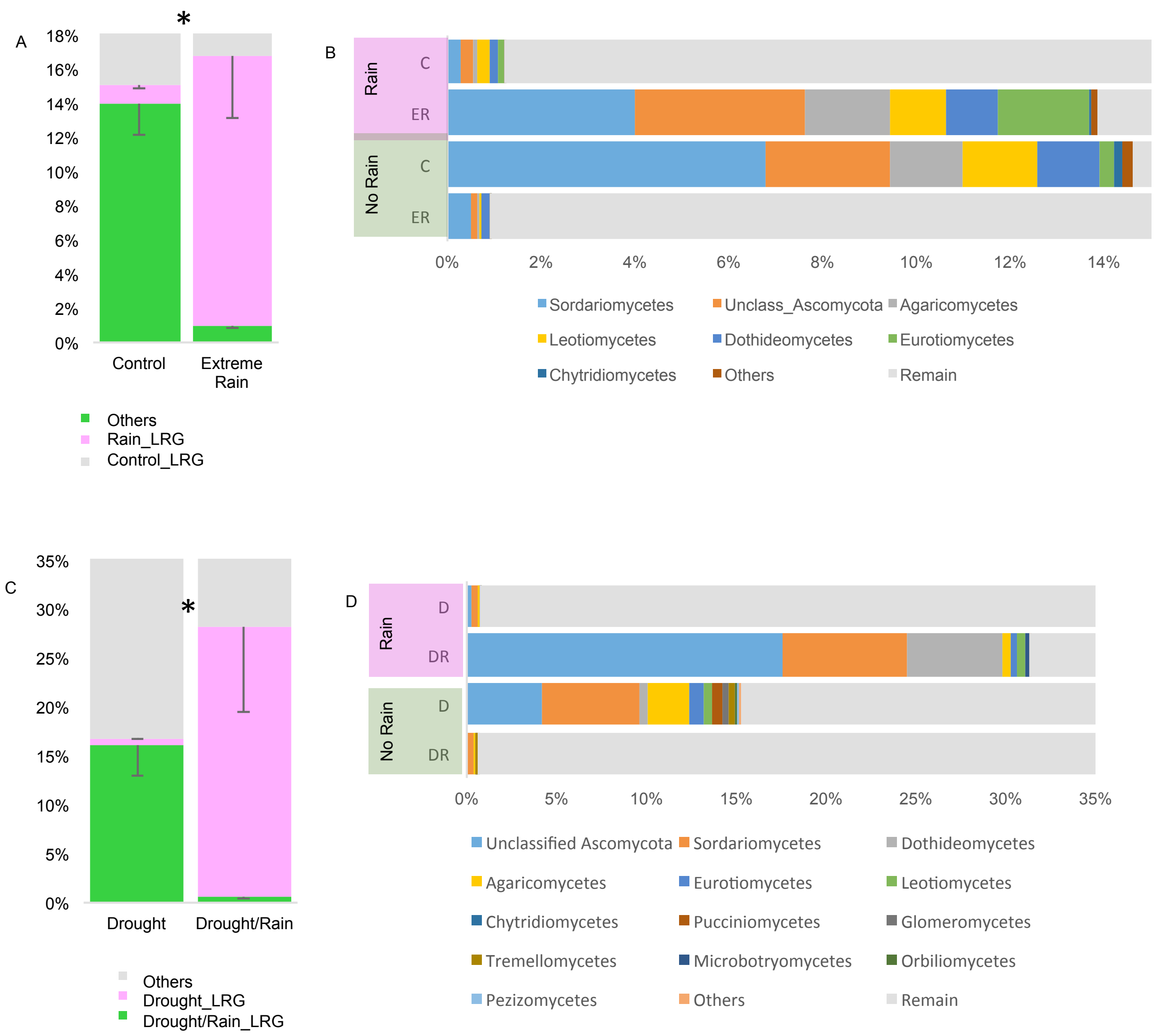

Figure S7: Legacy Response Groups of ITS were performed separately for Moist Control versus extreme re-wetting (rain in graphs) (A+B) and Drought versus drying and extreme re-wetting (rain in graph) (C+D). The relative abundance of OTUs that belong to the Response groups (A,C) and the taxonomical affiliation are presented (B,D). *indicates a difference between the treatments for the two legacy response groups at $P<0.05$.

Table S1: Summary table of the 16S rRNA gene amplicon samples. Samples in red were excluded as they have either too few counts, or very peculiar profiles with low richness and only unclassified OTUs. The samples with too low richness were likely contaminated, resulting in overrepresentation of very specific OTUs that were not present in all other samples. Although they display rather divergent profiles compared to other samples from the same group, samples in yellow were kept in the present analysis as they still cluster with other 16S profiles (see figure 2).

Samples	Treatment	Identification Letter	Legacy	Re-wetting	Sequences Number	Comment
9_43_16S	Drought	D1	No water	WHC 50%	119939	OK
10_44_16S	Drought	D2	No water	WHC 50%	139197	OK
14_46_16S	Drought	D3	No water	WHC 50%	50512	OK
17_49_16S	Drought	D4	No water	WHC 50%	145075	OK
21_50_16S	Drought	D5	No water	WHC 50%	248851	OK
33_58_16S	Drought	D6	No water	WHC 50%	128725	OK
35_59_16S	Drought	D7	No water	WHC 50%	156078	OK
52_68_16S	Drought	D8	No water	WHC 50%	267952	OK
12_45_16S	Drought/Extreme re-wet	DR1	No water	WHC 100%	141708	OK
15_47_16S	Drought/Extreme re-wet	DR2	No water	WHC 100%	186471	OK
16_48_16S	Drought/Extreme re-wet	DR3	No water	WHC 100%	272774	OK
25_54_16S	Drought/Extreme re-wet	DR4	No water	WHC 100%	109833	OK
27_56_16S	Drought/Extreme re-wet	DR5	No water	WHC 100%	127513	OK
31_65_16S	Drought/Extreme re-wet	DR6	No water	WHC 100%	139743	OK
36_60_16S	Drought/Extreme re-wet	DR7	No water	WHC 100%	111257	OK
45_62_16S	Drought/Extreme re-wet	DR8	No water	WHC 100%	63445	Too low richness,
5_73_16S	Moist Control	M1	WHC 50%	WHC 50%	149423	OK
24_53_16S	Moist Control	M2	WHC 50%	WHC 50%	116780	OK
44_61_16S	Moist Control	M3	WHC 50%	WHC 50%	103624	OK
46_63_16S	Moist Control	M4	WHC 50%	WHC 50%	14	Too few counts
48_57_16S	Moist Control	M5	WHC 50%	WHC 50%	176128	Outlier on ordination
49_66_16S	Moist Control	M6	WHC 50%	WHC 50%	149021	Outlier on ordination
59_70_16S	Moist Control	M7	WHC 50%	WHC 50%	58879	OK
61_71_16S	Moist Control	M8	WHC 50%	WHC 50%	60456	OK
4_41_16S	Extreme re-wet	R1	WHC 50%	WHC 100%	33568	Too low richness
22_51_16S	Extreme re-wet	R2	WHC 50%	WHC 100%	80364	OK
23_52_16S	Extreme re-wet	R3	WHC 50%	WHC 100%	105690	OK
26_55_16S	Extreme re-wet	R4	WHC 50%	WHC 100%	111036	OK
47_64_16S	Extreme re-wet	R5	WHC 50%	WHC 100%	121236	OK
50_67_16S	Extreme re-wet	R6	WHC 50%	WHC 100%	148344	Outlier on ordination
54_69_16S	Extreme re-wet	R7	WHC 50%	WHC 100%	198500	OK
63_72_16S	Extreme re-wet	R8	WHC 50%	WHC 100%	129014	OK

Table S2: Summary table of the ITS amplicon samples. Sample in red was excluded as it has too few counts. Samples in yellow have lower counts than others but still consistent profiles, although they lack rare OTUs. Samples in pink were kept, but they display extremely divergent profiles on ordination, clustering away from other ITS as outliers.

Samples	Treatment	Identification letter	Legacy	Re-wetting	Sequence number	Comment
9_43_ITS	Drought	D1	No water	WHC 50%	45312	OK
10_44_ITS	Drought	D2	No water	WHC 50%	24385	OK
14_46_ITS	Drought	D3	No water	WHC 50%	43766	OK
17_49_ITS	Drought	D4	No water	WHC 50%	138549	Outlier on ordination
21_50_ITS	Drought	D5	No water	WHC 50%	104701	OK
33_58_ITS	Drought	D6	No water	WHC 50%	48918	OK
35_59_ITS	Drought	D7	No water	WHC 50%	7183	Counts low
52_68_ITS	Drought	D8	No water	WHC 50%	156209	OK
12_45_ITS	Drought/ extreme re-wet	DR1	No water	WHC 100%	32744	OK
16_48_ITS	Drought/ extreme re-wet	DR2	No water	WHC 100%	82100	Outlier on ordination
25_54_ITS	Drought/ extreme re-wet	DR3	No water	WHC 100%	217	Too few counts
27_56_ITS	Drought/ extreme re-wet	DR4	No water	WHC 100%	76990	OK
31_65_ITS	Drought/ extreme re-wet	DR5	No water	WHC 100%	77889	OK
36_60_ITS	Drought/ extreme re-wet	DR6	No water	WHC 100%	17301	OK
45_62_ITS	Drought/ extreme re-wet	DR7	No water	WHC 100%	56666	OK
5_42_ITS	Moist Control	M1	WHC 50%	WHC 50%	50848	OK
24_53_ITS	Moist Control	M2	WHC 50%	WHC 50%	67074	OK
44_61_ITS	Moist Control	M3	WHC 50%	WHC 50%	25214	OK
46_63_ITS	Moist Control	M4	WHC 50%	WHC 50%	56651	OK
48_57_ITS	Moist Control	M5	WHC 50%	WHC 50%	83665	OK
49_66_ITS	Moist Control	M6	WHC 50%	WHC 50%	23972	OK
59_70_ITS	Moist Control	M7	WHC 50%	WHC 50%	32521	OK
61_71_ITS	Moist Control	M8	WHC 50%	WHC 50%	16772	OK
4_41_ITS	Extreme Re-wet	R1	WHC 50%	WHC 100%	59723	OK
22_51_ITS	Extreme Re-wet	R2	WHC 50%	WHC 100%	74875	OK
23_52_ITS	Extreme Re-wet	R3	WHC 50%	WHC 100%	6852	Counts low
26_55_ITS	Extreme Re-wet	R4	WHC 50%	WHC 100%	12951	OK
50_67_ITS	Extreme Re-wet	R5	WHC 50%	WHC 100%	61332	OK
54_69_ITS	Extreme Re-wet	R6	WHC 50%	WHC 100%	31855	OK
63_72_ITS	Extreme Re-wet	R7	WHC 50%	WHC 100%	104725	OK

Table S3: enrichment of OTUs at phylum level that decreased in relative abundance due to the drought legacy (LRG wet). LRG_OTU is the # of OTUs for that phylum in the LRG wet. All_OTU is the total number of OTUs for that phylum. LRG % is the % of OTU within the LRG wet that belonging to the specific phylum. All_% is the % of OTUs that belong to the Phylum. Log2 (ratio) is the enrichment test and P is the P value. A significant P value indicates that the OTUs belonging to the LRG are significantly enriched.

	LRG_OTU	All_OTU	LRG_%	All_%	log2(ratio)	P
Cyanobacteria/Chloroplast	37	144	14.341	0.625	4.521	< 0.0001
Chloroflexi	12	169	4.651	0.733	2.665	< 0.0001
Bacteroidetes	32	1412	12.403	6.127	1.018	< 0.0001
Verrucomicrobia	15	743	5.814	3.224	0.851	0.010
candidate_division_WPS-1	1	15	0.388	0.065	2.574	0.012
Euryarchaeota	1	25	0.388	0.108	1.837	0.032
candidate_division_WPS-2	1	96	0.388	0.417	-0.104	0.292
Parcubacteria	1	180	0.388	0.781	-1.011	0.601
Unclass_Bacteria	90	8652	34.884	37.541	-0.106	0.794
Proteobacteria	55	6131	21.318	26.602	-0.319	0.971
Chlamydiae	1	539	0.388	2.339	-2.593	0.984
Acidobacteria	5	1113	1.938	4.829	-1.317	0.987
Firmicutes	1	851	0.388	3.692	-3.252	0.999
Planctomycetes	5	1708	1.938	7.411	-1.935	1.000
Actinobacteria	1	1269	0.388	5.506	-3.828	1.000

Tables S4: Enrichment of OTUs at phylum level that increased in relative abundance due to the drought legacy (LRG dry). LRG_OTU is the # of OTUs for that phylum in the LRG dry. All_OTU is the total number of OTUs for that phylum. LRG % is the % of OTU within the LRG dry belonging to the specific phylum. All_% is the % of OTUs that belong to the Phylum. Log2 (ratio) is the enrichment test and P is the P value. A significant P value indicates that the OTUs belonging to the LRG are significantly enriched.

	LRG_OTU	All_OTU	LRG_%	All_%	log2(ratio)	P
Bacteroidetes	35	1412	20.96	6.87	1.61	<0.001
Thaumarchaeota	3	39	1.80	0.19	3.24	<0.001
Proteobacteria	65	6131	38.92	29.81	0.38	<0.001
Unclass_Archaea	1	20	0.60	0.10	2.62	0.01
Cyanobacteria/Chloroplast	3	144	1.80	0.70	1.36	0.03
Acidobacteria	10	1113	5.99	5.41	0.15	0.29
Gemmatimonadetes	1	193	0.60	0.94	-0.65	0.47
Firmicutes	4	851	2.40	4.14	-0.79	0.83
Verrucomicrobia	3	743	1.80	3.61	-1.01	0.86
Actinobacteria	6	1269	3.59	6.17	-0.78	0.90
Unclass_Bacteria	36	8652	21.56	42.07	-0.96	1.00

Table S5: enrichment of OTUs at phylum level that increased in relative abundance due to the drought legacy (LRG dry) or decreased in relative abundance due to drought legacy (LRG wet). LRG group describes the group where the enrichment is performed for. LRG_OTU is the # of OTUs for that phylum in the LRG dry. All_OTU is the total number of OTUs for that phylum. LRG % is the % of OTU within the LRG dry belonging to the specific phylum. All_% is the % of OTUs that belong to the Phylum. Log2 (ratio) is the enrichment test and P is the P value. A significant P value indicates that the OTUs belonging to the LRG are significantly enriched.

	LRG group	LRG_OTU	All_OTU	LRG_%	All_%	log2(ratio)	P
Ascomycota	LRG dry	48	1208	73.85	61.73	0.26	0.01
Basidiomycota	LRG dry	13	423	20.00	21.61	-0.11	0.56
Chytridiomycota	LRG dry	3	122	4.62	6.23	-0.43	0.59
Glomeromycota	LRG dry	1	204	1.54	10.42	-2.76	0.99
Ascomycota	LRG wet	37	1208	77.08	68.91	0.16	0.08
Basidiomycota	LRG wet	9	423	18.75	24.13	-0.36	0.76
Chytridiomycota	LRG wet	2	122	4.17	6.96	-0.74	0.66
